# Using environmental sampling to evaluate the effectiveness of decontamination methods to reduce detection of porcine epidemic diarrhea virus RNA on feed manufacturing surfaces

**DOI:** 10.1093/tas/txab121

**Published:** 2021-07-19

**Authors:** Mary B Muckey, Cassandra K Jones, Jason C Woodworth, Chad B Paulk, Steve S Dritz, Jordan T Gebhardt

**Affiliations:** 1 Department of Grain Science and Industry, College of Agriculture, Kansas State University, Manhattan KS 66506, USA; 2 Department of Animal Sciences and Industry, College of Agriculture, Kansas State University, Manhattan KS 66506, USA; 3 Department of Diagnostic Medicine/Pathobiology, College of Veterinary Medicine, Kansas State University, Manhattan, KS 66506, USA

**Keywords:** decontamination, porcine epidemic diarrhea virus, surface, swine, virus

## Abstract

Porcine epidemic diarrhea virus (PEDV) is a possible biological hazard in feed mills. If the virus enters a feed mill, it becomes widely distributed and is difficult to decontaminate from both feed contact and non-feed contact surfaces. The objective of this study was to evaluate a variety of liquid and dry decontamination treatments that could be used to reduce the amount of PEDV found on feed manufacturing surfaces. This experiment was designed as a 5 × 10 factorial with five different feed manufacturing surfaces and 10 decontamination treatments with three replicates of each combination. Surfaces included stainless steel, solid polyethylene, woven polypropylene tote bag, rubber, and sealed concrete coupons. One mL (1×10^5^ TCID_50_/mL) of stock PEDV was applied to each surface and allowed to dry completely for 60 min. Next, for decontamination requiring surface application, the application was performed and allowed 15 min contact time. The quantity of PEDV RNA was determined using quantitative reverse transcription PCR. A decontamination treatment × surface interaction was observed (*P* < 0.0001), indicating the efficacy of treatment is dependent upon the surface in which it is applied. Within the cement surfaces, the sodium hypochlorite resulted in the greatest (*P* < 0.05) cycle threshold **(Ct)** value, followed by formaldehyde which had a greater (*P* < 0.05) Ct value compared to remaining treatments. Within polyethylene, rubber, and stainless steel surfaces, the formaldehyde treated surfaces had the greatest Ct values (*P* < 0.05), followed by the sodium hypochlorite treatment, with other treatments all having lower Ct values (*P* < 0.05). For the woven polyethylene surfaces, the formaldehyde and sodium hypochlorite treatments had greater Ct values compared to all other treatments (*P* < 0.05). Additional research is necessary to identify the role of decontamination treatment on PEDV infectivity and develop methods for decontamination of feed manufacturing facilities.

## INTRODUCTION

It has been documented that contamination with bacterial (Torres et al., 2011; Burns et al., 2015; Magossi et al., 2019a; Magossi et al., 2019b) and viral pathogens (Greiner, 2016; Schumacher et al., 2017) can be identified within feed manufacturing facilities. Within the swine production industry in the United States, porcine epidemic diarrhea virus (PEDV) became an extremely important pathogen following introduction into North America in 2013. Several epidemiological investigations have provided evidence that contaminated feed or ingredients may have been involved with the spread of PEDV (Pasick et al., 2014; Bowman et al., 2015b; Aubry et al., 2017). Because of this, a significant body of research has focused on the potential for the feed supply chain to be involved with the transmission of livestock pathogens. Porcine epidemic diarrhea virus is a single-stranded, positive-sense RNA virus and is a member of the *Alphacoronavirus* genera (Jung and Saif, 2015). Clinical disease caused by PEDV is most severe in young piglets, with severe diarrhea and high levels of mortality (Stevenson et al., 2013). Inactivation of the virus has been shown to be possible with a number of virucidal disinfectants, including oxidizing agents, phenolic compounds, among others (Pospischil et al., 2002). The stability of PEDV on surfaces has been evaluated, and infectious virus was recovered between 2 and 15 days after inoculation depending on the surface and storage temperature (Kim et al., 2018).

When a biological agent such as PEDV is introduced into a feed manufacturing setting, the level of cross-contamination between batches of feed and environmental contamination within the facility has been shown to be widespread (Schumacher et al., 2017). It has been documented that decontaminating feed manufacturing facilities is very challenging, and the most effective means appear to be a combination of high pressure water, liquid decontamination, and thermal processes (Huss et al., 2015; Huss et al., 2017). However, the majority of feed manufacturing facilities are not designed with such decontamination methods in mind. Chemical disinfectants have shown some promise on reducing PEDV RNA on trailer surfaces (Bowman et al., 2015a), but there is limited information regarding their success on reducing viral RNA on feed manufacturing surfaces. Therefore, the objective of the current investigation was to evaluate the effectiveness of multiple decontamination treatment strategies to reduce the detection of PEDV genetic material on a number of surfaces commonly found within feed manufacturing facilities.

## MATERIALS AND METHODS

### Surface Preparation and Viral Inoculation

The experimental treatments were arranged as a 5 × 10 factorial with five different feed manufacturing surfaces, 10 decontamination treatments, and three replications of each combination. Surfaces included: (1) stainless steel (stainless steel type 316; Built-So-Well, Manhattan, KS); (2) solid polyethylene (Dura Bucket National Oats Co., Collinsville, IL); (3) rubber (RUB 220 belting; Maxi-Lift Inc., Addison, TX); (4) woven polypropylene tote bag (The MegaSack Corp., Magnolia, AR); and (5) concrete (Quikrete Co., Atlanta, GA) sealed with acrylic masonry sealer (Quikrete Co., Atlanta, GA). All surface coupons were equal in size (103.2 cm^2^), and were representative samples from larger scale manufacturing surfaces. Surface coupons were prepared by washing with soap, rinsing with distilled water, and cleaning with nucleic acid removing wipes (DNA AWAY, Molecular Bioproducts, San Diego, CA). After allowing surfaces to dry, surfaces were rinsed again with distilled water and sterilized in an autoclave at 121°C for 15 min. Next, 1 mL of cell culture-derived PEDV inoculum (USA/IN/2013/19338; 1 × 10^5^ TCID_50_/mL; initial cycle threshold (Ct) = 20.7) was applied to the surfaces and spread using a cell spreader to cover the entire area. Surfaces were allowed to dry for 60 min.

### Surface Treatment

After drying, respective treatment was applied to each coupon surface. One mL of liquid or 15 g of dry treatment was spread onto each surface for 15 min to allow for complete surface coverage. Immediately after dry treatment contact time was achieved, excess material was removed by sterile forceps and gently tapping twice. Chemical treatments included: (1) no decontamination (control); (2) untreated rice hulls; (3) rice hulls treated with formaldehyde-based product at 3.25 kg/tonne application rate (Sal CURB; Kemin Inc., Des Moines, IA; 30% formaldehyde and 10% propionic acid/methanol blend); (4) liquid formaldehyde-based product (Sal CURB; Kemin Inc., Des Moines, IA); (5) dry commercial benzoic acid and essential oil blend (VevoVitall and CRINA; 25:1 ratio of VevoVitall and CRINA; DSM Nutritional Products Inc., Parsippany, NJ; 96% benzoic acid and 4% essential oil blend); (6) liquid commercial food-grade sanitizer (DrySan Duo; Ecolab, St., Paul, MN; 10.98% isopropanol, 0.045% hydrogen peroxide, 0.016% alkyl dimethyl benzyl ammonium chloride, 0.007% dodecyl dimethyl ammonium chloride, and 0.005% dioctyl dimethyl ammonium chloride); (7) 3% dilution of liquid hydrogen peroxide (Intervention; Virox Technologies Inc., Ontario, Canada; 4.25% hydrogen peroxide); (8) 0.39% dilution of liquid quaternary ammonium glutaraldehyde (Synergize; Preserve International, Reno, NV; 26.0% alkyl dimethyl benzyl ammonium chloride and 7% glutaraldehyde); (9) 10% dilution of liquid sodium hypochlorite (The Clorox Company, Oakland, CA; 8.25% sodium hypochlorite); and (10) liquid medium chain fatty acid (MCFA) blend of hexanoic, octanoic, and decanoic acids (individual fatty acids guaranteed ≥ 98% purity, 1:1:1 wt:wt ratio; Sigma-Aldrich, St. Louis, MO).

### Sample Collection and Analysis

Following appropriate contact time, surfaces were then swabbed as previously described (Bowman et al., 2015a) to determine residual PEDV contamination using pre-moistened polyurethane foam tip environmental swabs in 5 mL of neutralizing broth (World Bioproducts LLC., Mundelein, IL). When surfaces were sampled, the entire 103.2 cm^2^ surface was sampled. Swabs were vortexed and PEDV was quantified using real-time, reverse-transcriptase PCR at the Kansas State University Molecular Diagnostics Development Laboratory as previously described (Schumacher et al., 2017). Data were reported from the laboratory as Ct value following standard operating procedures. Absence of detectable PEDV RNA (no detected RNA after 45 qRT-PCR cycles) was interpreted as a negative sample. The detection PEDV RNA (<45 qRT-PCR Ct), regardless of level, was interpreted as contaminated. Cycle threshold value is inversely related to quantity of detectable PEDV RNA, with lower Ct values having a greater quantity of detectable RNA.

### Statistical Analysis

Data were analyzed using a linear model fit using the GLIMMIX procedure of SAS version 9.4 (SAS Institute, Inc., Cary, NC) using individual surface sample as the experimental unit. In the event an analyzed sample did not have PEDV RNA detected after the laboratory research protocol cutoff of 45 PCR cycles, a value of 45 was assumed within the statistical analysis. Fixed effects in the model included the main effects of surface type, decontamination treatment, and the associated interaction. To further characterize the significant surface type × decontamination treatment interaction, simple effects were evaluated to evaluate the effect of decontamination treatment within each surface type and effect of surface type within each decontamination treatment as described by Stroup (2013). Simple effects were calculated using the SLICEBY option and pairwise differences within each simple effect were determined using the LINES option using a Bonferroni multiple comparison adjustment to control type I error rate. Main effects were evaluated using the LINES option using a Tukey–Kramer multiple comparison adjustment. Results were considered significant at *P* ≤ 0.05.

## RESULTS

There was evidence of a surface type × decontamination treatment interaction (*P* < 0.0001; Table 1) as well as main effect of surface type and decontamination treatment (both *P* < 0.0001; Table 2). Within the surfaces not treated (control), a greater amount of viral RNA (represented as lower Ct value) was detected on the rubber surface compared to cement, polyethylene, or woven polyethylene (*P* < 0.05). Furthermore, the cement and woven polyethylene surfaces had greater Ct values compared to stainless steel (*P* < 0.05). Within the surfaces exposed to rice hulls and rice hulls treated with 10% MCFA, the cement and woven polyethylene surfaces had greater Ct values compared to the polyethylene, rubber, and stainless steel surfaces (*P* < 0.05). Within the surfaces treated with formaldehyde, the cement surface had lower Ct value compared to all other surfaces (*P* < 0.05), with all three samples collected from the polyethylene, rubber, and stainless steel surfaces not having detectable RNA (Ct value of 45 assumed in the analysis for all samples) and two of three samples from the woven polyethylene surface not having detectable RNA. Within the surfaces exposed to the benzoic acid/essential oil blend, the cement and woven polyethylene surfaces had greater Ct values compared to the polyethylene, rubber, and stainless steel surfaces (*P* < 0.05). Within the surfaces treated with the food-grade sanitizer, the cement and woven polyethylene surfaces had greater Ct values compared to the polyethylene and rubber surface (*P* < 0.05), with the stainless steel surface being intermediate. Within the surfaces treated with the accelerated hydrogen peroxide, the cement and woven polyethylene surfaces had greater (*P* < 0.05) Ct values compared to rubber surfaces, with polyethylene and stainless steel surfaces being intermediate. Within surfaces treated with the quaternary ammonium/glutaraldehyde treatment, the cement surface had greater Ct values compared to the polyethylene, rubber, and stainless steel treatments (*P* < 0.05). Furthermore, the woven polyethylene surface had a greater Ct value compared to rubber (*P* < 0.05). Within surfaces treated with sodium hypochlorite, the cement and woven polyethylene surfaces had greater Ct values compared to polyethylene, rubber, and stainless steel. Finally, within surfaces treated with MCFA the cement surface had a greater Ct value compared to other treatments (*P* < 0.05), with no evidence of a difference among other treatments (*P* > 0.05).

**Table 1.** Interactive means of feed manufacturing surface type and decontamination method on cycle threshold quantification of porcine epidemic diarrhea virus RNA with environmental swabbing^1^,^2^,^3^

		Surface type				
Decontamination treatment	Application type	Cement	Polyethylene	Woven polyethylene	Rubber	Stainless steel
Control	N/A	27.5^a,z^	26.7^a,b,x^	28.3^a,w^	23.8^c,x^	24.6^b,c,x^
Rice hulls (RH)	N/A	31.2^a,x,y^	24.7^b,x^	28.9^a,w^	24.3^b,x^	24.5^b,x^
Formaldehyde RH^4^	N/A	30.3^a,x,y,z^	24.2^b,x^	28.5^a,w^	23.7^b,x^	24.5^b,x^
Formaldehyde^5^	Liquid	36.7^b,w^	45.0^a,v^	43.0^a,v^	45.0^a,v^	45.0^a,v^
Benzoic acid/essential oil^6^	Dry	30.6^a,x,y,z^	26.1^b,x^	29.8^a,w^	26.4^b,x^	26.3^b,x^
Food-grade sanitizer^7^	Dry	27.9^a,y,z^	24.9^b,x^	28.3^a,w^	24.7^b,x^	26.0^a,b,x^
Peroxide^8^	Liquid	27.7^a,z^	25.3^a,b,x^	27.8^a,w^	24.7^b,x^	27.2^a,b,x^
Quaternary ammonium/glutaraldehyde^9^	Liquid	31.7^a,x^	27.1^b,c,x^	29.7^a,b,w^	26.3^c,x^	27.3^b,c,x^
Sodium hypochlorite^10^	Liquid	40.4^a,v^	35.0^b,w^	43.0^a,v^	35.6^b,w^	37.1^b,w^
MCFA^11^	Liquid	31.1^a,x,y^	26.3^b,x^	27.4^b,w^	26.0^b,x^	26.0^b,x^

^1^ A total of 150 surface samples were used in a 5 × 10 factorial arrangement using five surface types, 10 chemical treatments, and three replicates per surface type × chemical treatment combination. One mL (1 × 10^5^ TCID_50_/mL) of stock PEDV was applied to each surface and allowed to dry completely for 60 min. The chemical treatments were then applied and allowed a 15 min contact time after which excess was removed. Surfaces were then swabbed using pre-moistened environmental swabs in 5 mL of neutralizing broth. Swabs were then analyzed using a quantitative, real-time, reverse-transcriptase PCR analytical procedure.

^2^ Treatment × surface, *P* < 0.0001. SEM = 0.72. All *F*-tests within simple effect: *P* ≤ 0.005. Interactive means sliced by chemical treatment and by surface type using Bonferroni multiple comparisons adjustment. Simple effects are characterized as follows: Within a row, means lacking a common lowercase letter (a,b,c) are different (*P* < 0.05). Within a column, means lacking a common lowercase letter (v,w,x,y,z) are different (*P* < 0.05).

^3^ When samples did not contain detectable PEDV genetic material following 45 cycles, a value of 45 was assumed in the statistical analysis. A Ct value of 45 was assumed for 3 of 3 samples for formaldehyde-treated polyethylene, rubber, and stainless steel; 2 of 3 samples formaldehyde-treated woven polyethylene and sodium hypochlorite-treated woven polyethylene; and 1 of 3 samples for sodium hypochlorite-treated cement.

^4^ Rice hulls treated with commercial formaldehyde (3.25 kg/tonne; Sal CURB; Kemin Industries, Des Moines, IA; 30% formaldehyde and 10% propionic acid/methanol blend).

^5^ Sal CURB (Kemin Industries, Des Moines, IA).

^6^ VevoVitall and CRINA (25:1 ratio; DSM Nutritional Products Inc., Parsippany, NJ; 96% benzoic acid and 4% essential oil blend).

^7^ DrySan Duo (Ecolab, St. Paul, MN; 10.98% isopropyl alcohol, 0.045% hydrogen peroxide, 0.016% alkyl dimethyl benzyl ammonium chloride, 0.007% dodecyl dimethyl ammonium chloride, and 0.005% dioctyl dimethyl ammonium chloride).

^8^ Intervention (3% dilution; Virox Technologies Inc., Ontario, Canada, 4.25% hydrogen peroxide).

^9^ Synergize (0.39% dilution; Preserve International, Reno NV; 26.0% alkyl dimethyl benzyl ammonium chloride and 7% glutaraldehyde).

^10^ Household bleach (10% dilution; The Chlorox Company, Oakland, CA; 8.25% sodium hypochlorite).

^11^ 1:1:1 blend of ≥ 98% purity C6:0, C8:0, and C10:0.

**Table 2.** Main effects of feed manufacturing surface type and decontamination treatment on cycle threshold quantification of porcine epidemic diarrhea virus RNA with environmental swabbing^1^,^2^

Main effect	Cycle threshold, Ct	SEM	*P* =
Surface		0.23	< 0.0001
Cement	31.5^a^		
Polyethylene	28.5^b^		
Woven polyethylene	31.4^a^		
Rubber	28.0^b^		
Stainless steel	28.8^b^		
Decontamination treatment		0.32	< 0.0001
Control	26.2^e^		
Rice hulls (RH)	26.7^d,e^		
Formaldehyde RH^3^	26.2^e^		
Formaldehyde^4^	42.9^a^		
Benzoic acid/essential oil^5^	27.8^c,d^		
Food-grade sanitizer^6^	26.3^e^		
Peroxide^7^	26.5^d,e^		
Quaternary ammonium/glutaraldehyde^8^	28.4^c^		
Sodium hypochlorite^9^	38.2^b^		
MCFA^10^	27.4^c,d,e^		

^1^ A total of 150 surface samples were used in a 5 × 10 factorial arrangement using five surface types, 10 chemical treatments, and three replicates per surface type × chemical treatment combination. One mL (1 × 10^5^ TCID_50_/mL) of stock PEDV was applied to each surface and allowed to dry completely for 60 min. The chemical treatments were then applied and allowed a 15 min contact time after which excess was removed. Surfaces were then swabbed using pre-moistened environmental swabs in 5 mL of neutralizing broth. Swabs were then analyzed using a quantitative, real-time, reverse-transcriptase PCR analytical procedure.

^2^ Treatment × surface, *P* < 0.0001. Interactive means presented in Table 1.

^3^ Rice hulls treated with commercial formaldehyde (3.25 kg/tonne; Sal CURB; Kemin Industries, Des Moines, IA; 30% formaldehyde and 10% propionic acid/methanol blend).

^4^ Sal CURB (Kemin Industries, Des Moines, IA).

^5^ VevoVitall and CRINA (25:1 ratio; DSM Nutritional Products Inc., Parsippany, NJ; 96% benzoic acid and 4% essential oil blend).

^6^ DrySan Duo (Ecolab, St. Paul, MN; 10.98% isopropyl alcohol, 0.045% hydrogen peroxide, 0.016% alkyl dimethyl benzyl ammonium chloride, 0.007% dodecyl dimethyl ammonium chloride, and 0.005% dioctyl dimethyl ammonium chloride).

^7^ Intervention (3% dilution; Virox Technologies Inc., Ontario, Canada, 4.25% hydrogen peroxide).

^8^ Synergize (0.39% dilution; Preserve International, Reno NV; 26.0% alkyl dimethyl benzyl ammonium chloride and 7% glutaraldehyde).

^9^ Household bleach (10% dilution; The Chlorox Company, Oakland, CA; 8.25% sodium hypochlorite).

^10^ 1:1:1 blend of ≥ 98% purity C6:0, C8:0, and C10:0 (Sigma Aldrich; St. Louis, MO).

Within main effect, means lacking a common lowercase letter are different (*P* < 0.05).

Within the cement surfaces, the sodium hypochlorite resulted in the greatest (*P* < 0.05) Ct value, followed by formaldehyde which had a greater (*P* < 0.05) Ct value compared to remaining treatments, and the control, formaldehyde-treated rice hulls, benzoic acid/essential oil, food-grade sanitizer, and peroxide-based disinfectant having the lowest (*P* < 0.05) Ct values. Within polyethylene, rubber, and stainless steel surfaces, the formaldehyde-treated surfaces had the greatest Ct values (*P* < 0.05), followed by the sodium hypochlorite treatment, with other treatments all having lower Ct values compared to both formaldehyde and sodium hypochlorite treatments (*P* < 0.05). For the woven polyethylene surfaces, the formaldehyde and sodium hypochlorite treatments had greater Ct values compared to all other treatments (*P* < 0.05).

When evaluating main effects, the cement and woven polyethylene surfaces had greater Ct values compared to all other surfaces (*P* < 0.05). The formaldehyde treatment resulted in the greatest Ct values (*P* <0.05), followed by sodium hypochlorite which resulted in greater Ct values compared to all remaining treatments (*P* <0.05). The quaternary ammonium/glutaraldehyde treatment had greater Ct values compared to control, untreated rice hulls, formaldehyde-treated rice hulls, food-grade sanitizer, and accelerated hydrogen peroxide (*P* < 0.05). Finally, the benzoic acid/essential oil blend had greater Ct values compared to control, formaldehyde-treated rice hulls, and food-grade sanitizer (*P* < 0.05).

## DISCUSSION

It had been documented that biological contamination can be detected within feed manufacturing facilities including both bacterial (Torres et al., 2011; Burns et al., 2015; Magossi et al., 2019a; Magossi et al., 2019b) and viral agents (Greiner, 2016; Schumacher et al., 2017). Additionally, when a biological agent such as PEDV is introduced into a feed manufacturing setting, the level of cross-contamination between batches of feed and environmental contamination within the facility has been shown to be widespread (Schumacher et al., 2017). Furthermore, the degree in which contamination was detected differed based on the surface type, including 44% of metal surfaces and 100% of plastic and rubber surfaces having PEDV detected following four sequences of initially uncontaminated feed manufactured after mixing a batch of PEDV-inoculated feed (Schumacher et al., 2017). This surface contamination was detected even after the sequenced feed itself did no longer contain PEDV genetic material (Schumacher et al., 2018). Additionally, a follow-up study demonstrated that dust generated following manufacturing of PEDV-inoculated feed can be infectious to naïve pigs (Gebhardt et al., 2018). Contamination of surfaces within a feed mill is widespread following pathogen introduction into the mill, but specific areas within the equipment are also an important area for cross-contamination between batches such as the interior of conveyors, bucket elevators, bins, and floors (Greiner, 2016; Schumacher et al., 2017). Thus, feed contact and non-feed contact surfaces are both important areas of contamination within feed mills that need to be appropriately decontaminated.

While the prevention of pathogen entry into the feed mill is a critical initial step in feed biosecurity including prevention of pathogen entry through ingredients, transportation, and people (Jones, 2011; Cochrane et al., 2016; Jones et al., 2019), procedures must be developed to effectively decontaminate feed manufacturing facilities. It has been documented that decontaminating feed manufacturing facilities is very challenging, and the most effective means appear to be removal of all feed debris and dust using high pressure water, liquid decontamination, and thermal processes (Huss et al., 2015; Huss et al., 2017). The majority of feed manufacturing facilities are not designed with such decontamination methods in mind. Therefore, it is important to develop effective decontamination methods that are safe, effective, and can be implemented in all feed manufacturing facilities. One such approach included evaluation of flushing pathogenic material through the feed manufacturing equipment (Gebhardt et al., 2018), but residual dust on non-feed contact surfaces would remain contaminated. The goal of the current investigation was to evaluate the effectiveness of multiple compounds to reduce the presence of PEDV genetic material on a number of surfaces commonly found within feed manufacturing facilities including rubber, polyethylene, concrete, and stainless steel as an initial step to the development of decontamination procedures.

One of the challenges associated with decontamination of feed manufacturing facilities is the wide variety of physical properties of the surfaces. A significant body of research has focused on methods to decontaminate aluminum livestock trailers (Bowman et al., 2015a; Baker et al., 2017; Holtkamp et al., 2017; Baker et al., 2018). Little information is available regarding the efficacy of disinfection on other surface types specifically for PEDV. In the current investigation when no chemical treatment was applied, the cement and woven polyethylene had less genetic material recovered compared to the rubber and stainless steel surfaces. Porosity has an impact on the recovery of virus from surfaces (Yeargin et al., 2015), but the surfaces used in the current experiment were relatively non-porous in nature. The current investigation demonstrates that there is a difference in the quantity of genetic material recovered from different surfaces present within feed manufacturing. The reasoning behind the difference in quantification is not clear. It could be due to the physical properties of the surface and potential impact on viral stability, or could simply be a function of differences in viral recovery due to the before mentioned differences in surface properties.

Antimicrobial agents used for decontamination of surfaces may vary in type and concentration due to intended use, safety, and efficacy profiles. For many disinfectants, the greatest efficacy is observed in the absence of organic material. Thus, effective decontamination often requires physical cleaning, chemical treatment, rinsing with water, and complete drying. Commonly used antimicrobials for PEDV include aldehydes such as glutaraldehyde, oxidizing agents such as chlorine-based halogens and peroxygen compounds, phenols, and quaternary ammonium compounds. Additionally, research has been conducted evaluating the use of various compounds in swine feed and ingredients and has demonstrated that MCFA (Cochrane et al., 2016; Cochrane et al., 2020; Gebhardt et al., 2020) and formaldehyde (Dee et al., 2014; Cochrane et al., 2020) have efficacy at reducing quantity of PEDV genetic material and reducing infectivity when incorporated into swine feed and ingredients. Furthermore, the combination of benzoic acid and essential oils has been evaluated and demonstrated a reduction of PEDV genetic material (Gebhardt et al., 2019). Our study was the first to evaluate these compounds for the efficacy of surface decontamination.

In the current investigation, the efficacy of genetic material reduction was dependent on the surface being evaluated. In general, formaldehyde and sodium hypochlorite were most effective at reducing detection of PEDV. It is important to understand that the efficacy of decontamination from a practical sense is eliminating infectivity potential, where the outcome measured in the current investigation is detection of genetic material. It is possible that the formaldehyde and sodium hypochlorite were more effective at altering genetic material to make it undetectable compared to other disinfectants, while other antimicrobials may be equally as effective at preventing infection compared to formaldehyde or sodium hypochlorite without reducing the detectability of genetic material to the same extent.

The current study evaluated the impact of multiple decontamination treatments on detection of PEDV genetic material as measured using PCR and quantified by Ct values. While this method is rapid, cost effective, and sensitive for the presence of genetic material, it does not give any indication of infectivity, which is a limitation (Bowman et al., 2015a). In the current study, we performed all analysis using PCR techniques, and did not evaluate infectivity. The current virus isolation techniques make it difficult to evaluate infectivity potential for the cell culture-derived virus used in this experiment, and a bioassay is the preferred means to evaluate infectivity (Holtkamp et al., 2017). While bioassays have been commonly performed due to this (Bowman et al., 2015a; Baker et al., 2017; Holtkamp et al., 2017; Baker et al., 2018), the current investigation did not incorporate bioassay in order to increase the number of surface types and disinfectants evaluated which would have made full characterization of infectivity characteristics impractical. This does not minimize the importance of biological assays to evaluate infectivity potential, but does allow for preliminary data on efficacy to be generated for which further investigation can be performed.

When considering application of antimicrobial agents for surface decontamination, it is important to consider a variety of characteristics such as efficacy and safety for personnel and the environment. The use of all products should follow the label recommendations and safety precautions. The interaction of the antimicrobial agent with the surfaces of interest is very important, especially with routine product application. Many disinfectants such as acids, alkalis, and sodium hypochlorite are known to be corrosive on various surface types (Dvorak, 2008; Rutala et al., 2019). Due to this risk of damage to equipment and surfaces, the surface type, disinfectant properties, and frequency of antimicrobial agent application must be considered when incorporating surface disinfection procedures into a biosecurity program.

An important component of the current investigation was to evaluate the effectiveness of dry antimicrobial agents compared to liquid applied antimicrobial agents. It is plausible that the effectiveness of liquid-applied antimicrobials would be greater than dry-applied antimicrobials due to increased surface contact. In the current study, the dry antimicrobials included the combination of benzoic acid and essential oils and the food-grade sanitizer. Both compounds did not work as well as multiple other antimicrobials applied in liquid form including formaldehyde and sodium hypochlorite which would support the hypothesis that liquid application results in greater efficacy. However, it is important to acknowledge that different concentrations of both the liquid and dry applied antimicrobials might result in a different response. The use of antimicrobials applied in dry form would be much easier to implement in feed mills compared to liquid application, but the current investigation demonstrates the liquid application is more effective for the products, concentrations, and surfaces evaluated. Additional research is necessary to further understand the potential differences in efficacy between dry and liquid application of antimicrobial agents.

## CONCLUSION

In summary, liquid formaldehyde and liquid sodium hypochlorite were the most effective chemical treatments, but their application is limited due to their liquid state and potential corrosiveness in animal food manufacturing. Surface type can also influence PEDV mitigation strategies, particularly on rubber belting in bucket elevators or stainless steel, which can be more challenging to decontaminate in animal food facilities. Additional research is necessary to identify the role of decontamination strategies within a feed manufacturing setting on PEDV infectivity using biological assays and to develop decontamination methods for both animal feed contact and non-contact surfaces within feed manufacturing surfaces.
